# Effects of Glucose and Homogenization Treatment on the Quality of Liquid Whole Eggs

**DOI:** 10.3390/foods11162521

**Published:** 2022-08-20

**Authors:** Wei Hu, Yong Wu, Hongbing Chen, Jinyan Gao, Ping Tong

**Affiliations:** 1 State Key Laboratory of Food Science and Technology, Nanchang University, Nanchang 330047, China; 2 College of Food Science & Technology, Nanchang University, Nanchang 330047, China; 3 Sino-German Joint Research Institute, Nanchang University, Nanchang 330047, China

**Keywords:** liquid whole eggs, glucose, homogenization, processing property, functional properties, protein structure, particle size distribution, turbidity, protein analysis, protein unfolding-aggregation

## Abstract

To investigate the effect of glucose on the protein structure, physicochemical and processing properties of liquid whole eggs (LWE) under homogenization, different concentrations of glucose (0.01, 0.02, 0.04, 0.08 g/mL) were added into LWE, followed by homogenizing at different pressures (5, 10, 20, 40 MPa), respectively. It was shown that the particle size and turbidity of LWE increased with the increase in glucose concentration while decreasing with the increase in homogenization pressure. The protein unfolding was increased at a low concentration of glucose combined with homogenization, indicating a 40.33 ± 5.57% and 165.72 ± 33.57% increase in the fluorescence intensity and surface hydrophobicity under the condition of 0.02 g/mL glucose at 20 MPa, respectively. Moreover, the remarkable increments in foaming capacity, emulsifying capacity, and gel hardness of 47.57 ± 5.1%, 66.79 ± 9.55%, and 52.11 ± 9.83% were recorded under the condition of 0.02 g/mL glucose at 20 MPa, 0.04 g/mL glucose at 20 MPa, and 0.02 g/mL glucose at 40 MPa, respectively. Reasonably, glucose could improve the processing properties of LWE under homogenization, and 0.02 g/mL–0.04 g/mL and 20–40 MPa were the optimal glucose concentration and homogenization pressure. This study could contribute to the production of high-performance and stable quality of LWE.

## 1. Introduction

Liquid eggs are a type of egg product served in liquid form by removing the eggshell, which are generally homogenized and pasteurized before being packaged. Compared with traditional shell eggs, liquid eggs show the strengths of high safety, convenience, and compound diversity [1,2]. Liquid whole eggs (LWE) are a type of liquid egg, and they are mostly used in the raw material supply of bakery products. Meanwhile, the pleasant taste of processed foods benefits from the promising and diversified processing properties of LWE [3]. In industrial production, homogenization has almost become a must for egg product manufacturers. LWE may contain sedimentation of heavy or coagulated particles after pasteurization. The particles and fat globules can be ruptured by homogenization and a more stable LWE with a uniform composition could be produced [4]. Additionally, studies have proved that homogenization could increase the foaming capacity of liquid eggs and prevent decomposition during storage in egg products [5]. When the fluid is being homogenized, liquids are propelled to pass through a cabined valve. The structure and physicochemical properties of proteins were potentially modified by the shear force and rising temperature [6,7], and the protein unfolding and aggregation occurred frequently [8,9].

According to different product requirements, sugars, salts, and/or other ingredients are added to the LWE. Previous studies have shown the addition of sugars might have an impact on the interactions, such as hydrophobic and electrostatic interactions between proteins and other molecules [10]. Glucose, one of the simplest monosaccharides, is widely found in various processed and unprocessed foods. It is also the most abundant sugar in egg whites, accounting for 98% of their carbohydrates [11]. The temperature rises during homogenization, creating favorable conditions for the Maillard reaction between glucose and proteins [12]. In addition, there are many hydrogen bonds in the secondary structures of proteins. As a typical polyhydroxy aldose, glucose may interact with egg proteins through hydrogen bonds during homogenization, thus affecting protein structure [13,14]. How to generate high-performance and stable-quality products is an urgent issue in LWE production. Actually, a considerable number of studies have proved that processing properties are closely related to the structure and physicochemical properties of food proteins [12,15]. In this study, the effect of glucose on the quality of LWE under the homogenization conditions of industrial production was studied. Glucose was added according to the common requirements of enterprises; the amounts were 0.01, 0.02, 0.04, and 0.08 g/mL, respectively. LWE was homogenized under different pressures (5, 10, 20, and 40 MPa), which were simulated the conditions of industrial production. All measurements were repeated in triplicate and a multivariate ANOVA with SPSS software was performed. This study aimed to optimize the glucose addition and homogenization conditions so as to provide scientific and theoretical information for liquid egg manufacturers.

## 2. Materials and Methods

### 2.1. Materials

Fresh chicken eggs were procured from a supermarket named Wang Zhong Wang (Lao Nan Gou, Nanchang, Jiangxi, China). Glucose was purchased from XiLong Scientfic Co., Ltd. (Guangzhou, Guangdong, China). Phosphate buffered saline (PBS), sodium dodecyl sulfate (SDS), tris-(hydroxymethyl)-aminomethane (Tris), and ethylene diamine tetraacetic acid (EDTA) were obtained from Beijing Solarbio Technology Co., Ltd. (Beijing, China). Bovine serum albumin (BSA), 8-Anilino-1-naphthalenesulfonic acid (ANS) and 5,5′-Dithiobis-(2-nitrobenzoic acid) (DTNB) were obtained from Sigma Chemicals, (St. Louis, MO, USA). Coomassie brilliant blue G250 was supplied from Wuhan Seville Biological Technology co., ltd (Wuhan, Hubei, China). The vegetable oil was procured from COFCO. ELISA plates were purchased from Shenzhen Jin Can Hua Industrial Co., Ltd. (Shenzhen, Guangdong, China).

### 2.2. Sample Preparation

Fresh chicken eggs were washed first and then removed the eggshells. The LWE was collected and stirred magnetically at 300–500 r/min for 30 min, followed by filtering to remove egg ribbons. Glucose with different concentrations (0.01 g/mL, 0.02 g/mL, 0.04 g/mL, and 0.08 g/mL) were added into the LWE, respectively, followed by pouring into a homogenizer and homogenizing for 45 s at 5 MPa, 10 MPa, 20 MPa, and 40 MPa, respectively.

### 2.3. Turbidity Measurement

Turbidity could be used to investigate the protein aggregation in samples. The turbidity was measured according to the method of Liu et al. [16] with some modifications. The samples were diluted using 0.01 mol/mL PBS (pH = 7.4) to a ratio of 1:200. Then, 200 μL of diluted sample was added into each well of a 96-well microtiter plate, and the absorbance was measured at 600 nm.

### 2.4. Particle Size Measurement

Particle size can be used to explore the distribution of particles in liquid samples. The measurement of particle size was on the bases of the methods of Maghamian et al. [17] and conducted by a Malvern Mastersizer 3000 (Malvern Instruments Ltd., Worcestershire, UK). The obscuration range was 10–15%. The particle size was indicated as the droplet volume-average diameter D[4,3]:

(1) $$D[4,3]=\sum_{i}n_{i}d_{i}^{4}/\sum_{i}n_{i}d_{i}^{3}$$

The d_i_ is the diameter of the droplet and n_i_ is the amount. The changing regularity of particle size was calculated according to the drawn particle size distribution diagram.

### 2.5. Solubility Determination

Solubility is one of the important characteristics of egg production, which impacts the processing properties of proteins. The measurement of protein solubility was modified according to the method of Wu [8]. Coomassie brilliant blue method was used to determine the protein concentration in samples before and after centrifugation. Uncentrifuged samples were diluted with PBS at a ratio of 1:1000 (*v*/*v*), the supernatant of the centrifuged samples (for 4 min at 10,000× *g* and 4 °C) were diluted by the same method. Similarly, 20 μL uncentrifuged (A_0_) and centrifuged sample (A_1_) were separately mixed with 200 μL Coomassie Brilliant Blue G-250 solution. Finally, they were kept in the darkness for 10 min, followed by measuring their absorbances at 595 nm. The solubility was identified by the ratio of the protein concentration in supernatant before (A_0_) and after the centrifugation (A_1_).

### 2.6. Intrinsic Fluorescence Spectroscopy Determination

The chromophores on aromatic amino acid residues such as tryptophan and tyro-sine were particularly sensitive to changes in environmental polarity. The intrinsic fluorescence spectroscopy could show the tertiary structure change process of protein [18]. The sample was diluted to 1 mg/mL by PBS (1:120) and its fluorescence intensity was scanned under the following conditions: excitation and emission wavelengths were separately 250 nm and 270–400 nm, light step 1 nm, test time for 200 ms, slit width 5 nm.

### 2.7. Surface Hydrophobicity Determination

Surface hydrophobicity is closely related to foaming and emulsifying properties of proteins. The determination of surface hydrophobicity was performed by the method described by Mir [19]. The sample was diluted to 1 mg/mL by PBS (1:120). Then 200 μL of the sample and 40 μL of 5 mM ANS solution were mixed in 96-well microtiter plates at room temperature, followed by detecting the fluorescence intensity after the mixture were placed in the dark for 1 h. The excitation wavelength was 350 nm and the emission wavelength range was 400~700 nm, where the scan speeds, the optical path. the slit width and the response time were 120 nm/min, 1 cm, 5 nm, and 0.5 s, respectively.

### 2.8. Free Sulfhydryl Content Determination

Free sulfhydryl content could be used for investigating the changes in protein structure. The free sulfhydryl content was measured using the methods of Zhang [20] with some modifications. A total of 500 μL sample was evenly dispersed in 5 mL SDS-Tris-Gly buffer. Then, 4 mL of the prepared sample was mixed with 40 μL of Ellman reagent (0.04 g DTNB, 10 mL Tris-Gly buffer), followed by being kept in darkness for 30 min, and it was centrifugated for 20 min (8000× *g*, 4 °C). The free sulfhydryl content was calculated by Formula (2). The SDS-Tris-glycine buffer was the blank control.

Free sulfhydryl content: SH (μmol/g) = 75.53 × A_412_ × D/C (2)

75.53 was calculated from unit conversion (10^6^/1.36 × 10^4^ mol/L^−1^⋅cm^−1^), C is the concentration of protein sample, the unit is mg/mL, A_412nm_ = absorbance of sample added Ellman reagent-absorbance of the sample without Ellman reagent, D is the dilution factor of the sample, D = 10, C = 10.

### 2.9. Foaming Properties

The foaming properties were measured according to the previous study with some modifications [21,22]. The 4 mL sample was diluted with 36 mL PBS buffer (0.01 M). The diluted samples were poured into the same measuring cylinder and the foam heights before and after being whisked at 10,000× *g* for 30 s were recorded. Continue standing for 30 min, the foam height H_2_ was measured. The tests were repeated in triplicate.

(3) $$\mathrm{Foaming}\;\mathrm{capacity},\mathrm{FC}=\left(H_{1}-H_{2}\right)/H_{0}\times 100\%$$

*H*_0_ is the original height of the sample before stirring.

*H*_1_ is the starting height of foam after stirring.

(4) $$\mathrm{Foaming}\;\mathrm{stability},\mathrm{FS}=H_{2}/H_{1}\times 100\%$$

*H*_2_ is the foam height after stirring for 30 min.

### 2.10. Emulsifying Properties

The emulsifying properties were determined using the method described by Mozafarpour [23]. The samples were diluted with PBS to 10% (*v*/*v*). Vegetable oil was added at a ratio of 3:1 (*v*/*v*), and the samples were agitated by the high-speed disperser for 2 min at 8000× *g*. In total, 50 μL of the sample pipetted from the bottom of the container was mixed in 5 mL of 0.1% SDS to obtain the emulsion. In total, 200 μL of the emulsion was added to each well in a 96-well microtiter plate, and the absorbance measured at 500 nm wavelength was expressed as A_0_. Then the sample was let stand for 10 min and the absorbance measured at 500 nm wavelength was expressed as A_10_. The emulsifying activity index and emulsifying stability index are calculated according to the following formula:

(5) $$\mathrm{emulsifying}\;\mathrm{activity}\;\mathrm{index},\mathrm{EAI}=\frac{2\times 2.303\times A_{0}\times D}{(1-\varphi )\times C\times 10^{4}}$$

C represents the sample concentration before emulsification (g/mL). φ represents the oil volume fraction of the emulsion (*v*/*v*), where it is 0.25. D is the dilution factor.

(6) $$\mathrm{emulsifying}\;\mathrm{stability}\;\mathrm{index},\mathrm{ESI}=\frac{A_{10}}{A_{0}}\times 100\%$$

A_0_ is the absorbance measured at 500 nm 0 min after emulsification.

A_10_ is absorbance measured at 500 nm 10 min after emulsification.

### 2.11. Gelling Properties

LWE samples were made into thermal-denatured gels by water bath heating for 30 min at 90 °C. The hardness of the gel was determined by using a universal TA. XT plusC Texture Analyser (Stable Micro Systemsc Ltd., Godalming, Surrey, UK). Each gel was compressed twice to half of its original height at a crosshead speed of 1 mm/s with a trigger point load of 5 g, and the period was 5 s. The hardness was the peak force (N) required amid the initial compression cycle [24]. The water holding capacity (WHC) was measured by the method of Khemakhem with some modifications [25]. The gel samples were centrifuged at 4000× *g* for 10 min, and the weights of the gel before and after centrifugation were recorded to calculate the WHC as follows:

(7) $$\mathrm{Water}\;\mathrm{holding}\;\mathrm{capacity},\mathrm{WHC}=\frac{m_{1}}{m_{0}}\times 100\%$$

m_0_ is the gel weight before centrifugation.

m_1_ is the gel weight after centrifugation.

### 2.12. Statistical Analysis

All measurements were repeated in triplicate. A multivariate ANOVA and Duncan’s multiple comparisons with SPSS software (Version 24.0, SPSS Inc., Chicago, IL, USA)) were performed to determine whether statistical differences existed between different experimental groups. Figures were plotted by Origin software (Version 9.0, OriginLab, Northampton, MA, USA).

## 3. Results and Discussion

### 3.1. Structure Analysis of Protein in LWE

#### 3.1.1. Intrinsic Fluorescence Spectroscopy

**Figure 1:**
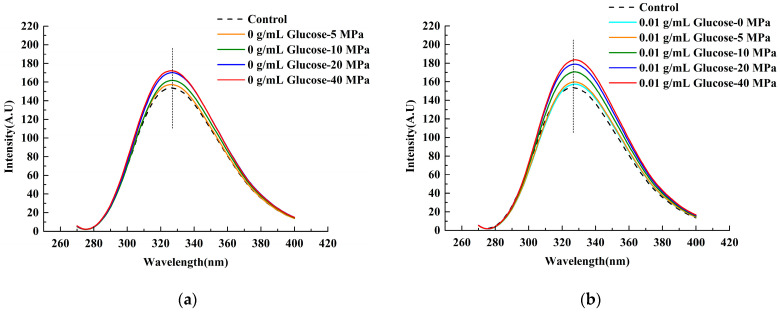

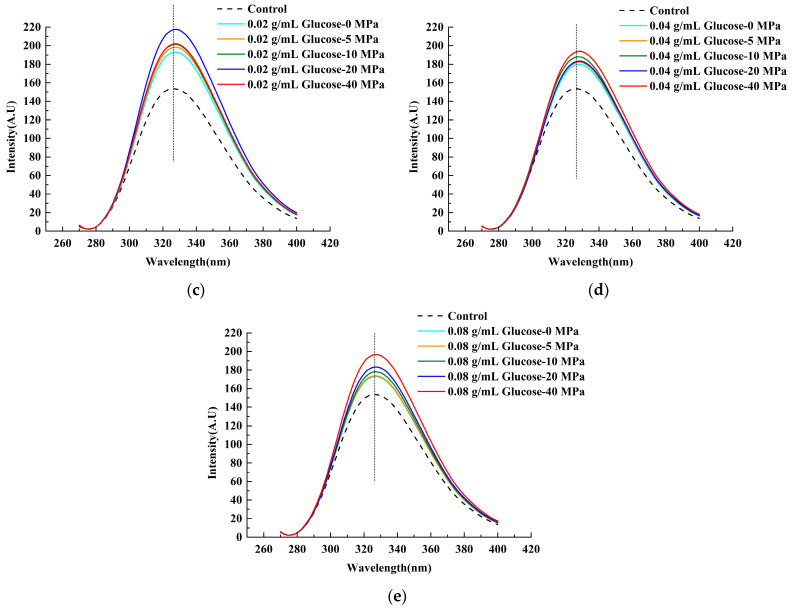
The intrinsic fluorescence spectra of LWE under different homogenization pressure treatment and glucose concentrations. (**a**–**e**) Presented the intrinsic fluorescence spectra of the LWE after adding 0 g/mL, 0.01 g/mL, 0.02 g/mL, 0.04 g/mL, and 0.08 g/mL glucose in turn.

The chromophores on aromatic amino acid residues such as tryptophan and tyrosine were particularly sensitive to changes in environmental polarity, and they could show the tertiary structure change process of protein [18]. The endogenous fluorescence emission spectra of each LWE sample are shown in Figure 1. The fluorescence intensity of the control was 154 ± 2.77 AU. In comparison with control, λmax shifted indistinctively while the intensity showed an increase in varying degrees and reached the maximum value of 218.9 ± 12.34 AU under the condition of 0.02 g/mL glucose at 20 MPa. On the one hand, when the homogenous pressure was from 0 MPa to 40 MPa, the hydrophobic bonds were broken by a stronger shear force, and the protein structure was more unfolded [26], which made the tryptophan residues in the internal hydrophobic structure be exposed to a polar environment [27,28]. Moreover, the fluorescence intensity increased at first and then decreased due to the effect of glucose, and the maximum one was at 0.02 g/mL of glucose, indicating that the protein structure in LWE would be unfolded when glucose was added at 0.01–0.02 g/mL. However, glucose at excessive addition (0.04–0.08 g/mL) might be grafted on the tryptophan and tyrosine residues, which affects the vibration of the tryptophan group, resulting in fluorescence quenching [29,30].

#### 3.1.2. Surface Hydrophobicity

**Figure 2:**
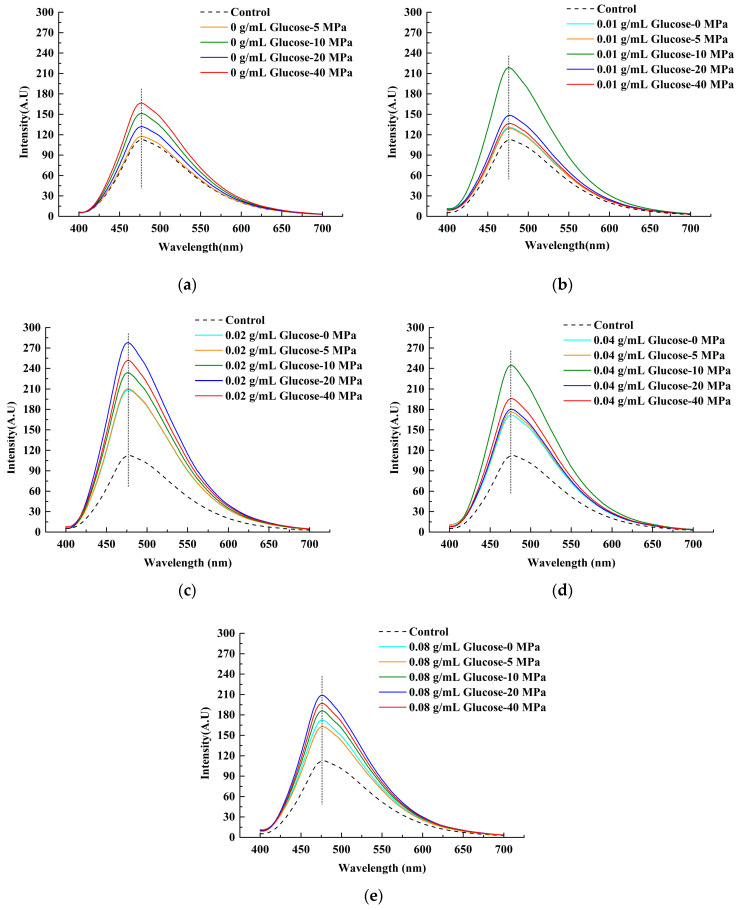
The exogenous fluorescence spectra of LWE under different homogenization pressure treatment and glucose concentrations. (**a**–**e**) Presented the exogenous fluorescence spectra of the LWE after adding 0 g/mL, 0.01 g/mL, 0.02 g/mL, 0.04 g/mL, and 0.08 g/mL glucose in turn.

ANS could be bonded to the hydrophobic groups of the protein structure [31], so it is usually applied to measure the protein surface hydrophobicity [32,33]. The exogenous fluorescence spectrum of LWE was shown in Figure 2. There was no obvious blueshift or redshift phenomenon presented in the emission spectrum as the homogenization pressure and glucose concentration both rose. The fluorescence intensity increased remarkably, indicating that the surface hydrophobicity of the samples could be enhanced by both homogenization and glucose, and the maximum was 0.02 g/mL of glucose at 20 MPa. When the pressure increased from 0 MPa to 40 MPa, the fluorescence intensity rose to the highest point at 20 MPa and then showed a slight decline at 40 MPa, where the trend was similar to the results of Yu’s study [34]. With the increase in the homogenization pressure, some aggregations became smaller. Moreover, the hidden hydrophobic groups were gradually exposed due to turbulence and shear. However, it should be noted that slight protein aggregation might be triggered by excessive pressure [34].

An inverted V-shaped change trend of the fluorescence intensity was found with the increase in glucose concentration, and the highest point was at 0.02 g/mL, which was consistent with the endogenous fluorescence spectra (Figure 1). Collectively, the surface hydrophobicity was all higher than the control. A previous study had proved that the addition of polysaccharides can enhance the hydrophobicity of the soy protein [35], and our results tended to be consistent with it, although the protein types were different. The inverted V-shaped change trend in surface hydrophobicity was attributed to the glycosylation that occurred in LWE under homogenization. In the early stages of glycosylation, more hydrophobic groups might be exposed. While in the later stage, large amounts of -OH of glucose were grafted onto the protein, and glucose may interact with egg proteins through a hydrogen bond, leading to a rapid decrease in surface hydrophobicity [14,36].

#### 3.1.3. Free Sulfhydryl

**Figure 3:**
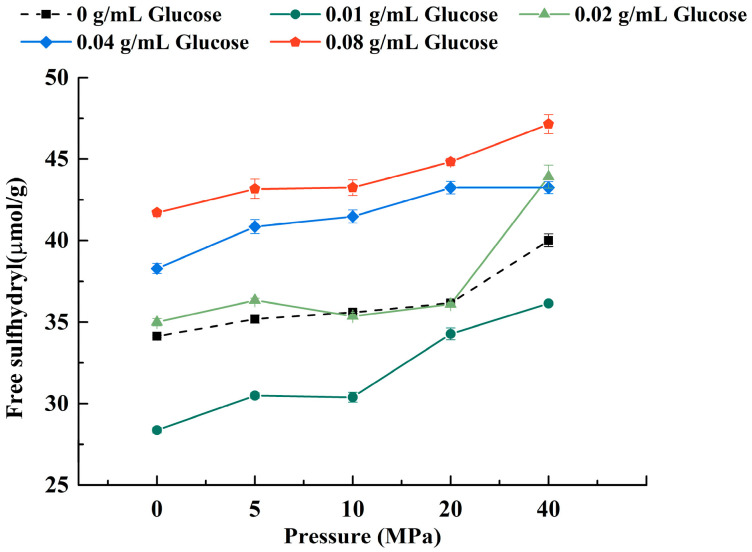
The free sulfhydryl content of LWE under different homogenization pressure treatment and glucose concentrations.

It was found that glucose lower than 0.02 g/mL would reduce the free sulfhydryl content in LWE (Figure 3), which implied that more disulfide bonds were formed in the protein. With the continuous increase of concentration, especially at 0.04 g/mL, the free sulfhydryl groups in LWE increased, which might be related to the reducibility of glucose, leading to the reduction of disulfide bonds in the protein structure [37]. Moreover, protein structure was unfolding after adding lower concentration glucose, and part of embedded sulfhydryl groups were exposed according to the fluorescence spectroscopy results (Figure 1 and Figure 2). Additionally, more free sulfhydryl groups in LWE were formed by homogenization. Some researchers reported that the disulfide could be broken by mechanical force. Based on the previous work and the analysis of changes in protein structure in this study, it was shown that disulfide bonds were partially enclosed in the tertiary structure of the protein [38], and it was consequently hard to contact with the oxidizing agent or reducing agent [39]. However, the observation of intrinsic and exogenous fluorescence spectrums (Figure 1 and Figure 2) implied that the tertiary structure of the protein was destroyed by homogenization and exposed to the surface [40], which made the disulfide bonds more easily reduced to -SH by glucose molecules.

### 3.2. Physicochemical Properties of LWE

#### 3.2.1. Particle Size

**Figure 4:**
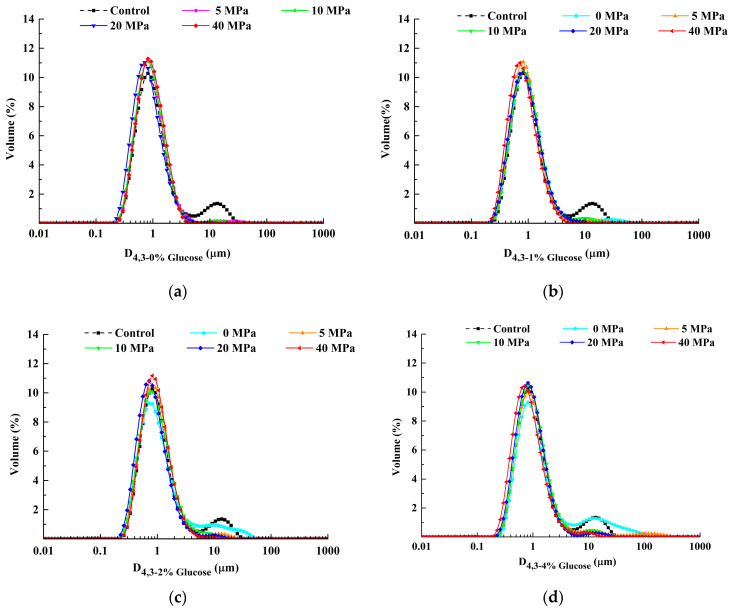

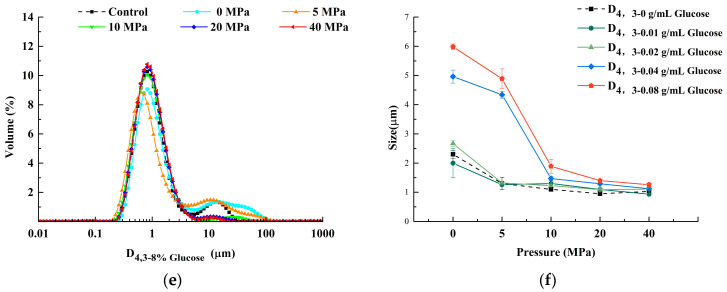
The particle size of LWE under different homogenization pressure treatment and glucose concentrations. Note: (**a**–**e**) presented the particle size distribution of the LWE after adding 0 g/mL, 0.01 g/mL, 0.02 g/mL, 0.04 g/mL, and 0.08 g/mL glucose in turn. (**f**) Presented the results of average volume particle size D[4,3] of LWE under different homogenization pressure treatment and glucose concentration.

The particle size distribution (PSD) and volume—average particle size of LWE were investigated under different processing conditions. All figures distinctly evinced that LWE had a bimodal distribution, with one distinct size distribution peak at about 1 μm and the other at about 10 μm (Figure 4a). The volume fraction of the particle size at around 1 μm gradually decreased when the glucose concentration increased, while it increased at around 10 μm. Especially when the glucose concentration was 0.08 g/mL, the fraction of 10 μm particles was the highest. Regarding homogenization, the fraction of 10 μm particles decreased significantly (*p* < 0.05) when pressure was 5–10 MPa. By contrast, LWE almost showed a single peak distribution as pressure was 20–40 MPa. The particle components in LWE showed a more uniform distribution with higher pressure, which gave rise to the depolymerization of aggregations around 10 μm [41], and even decreased the diameter of some protein particles [42]. The actual size of a single droplet in the LWE could be reflected by D[4,3] (Figure 4f). The volume-average particle size of the control was 2.3 ± 0.06 μm. Under different homogenization pressures, the particle size of LWE was decreased to different degrees and dropped to the lowest size (0.934 ± 0.01 μm) under the treatment condition of 0.01 g/mL glucose-40 MPa. Some structures of biomacromolecules, such as proteins and lipids in LWE, might be destroyed by the high-speed shearing force and turbulence induced by homogenization [43], resulting in a reduction in particle size. A similar decline in D[4,3] caused by homogenization was observed in the previous studies on scallop protein [8] and myofibrillar protein [44]. Additionally, it was manifested that homogenization led to the change from a bimodal distribution to a unimodal distribution, and the volume of the particle size at around 10 μm was significantly reduced, which also explained the reduction of D[4,3] effectively. A lower concentration (0.01–0.02 g/mL) of glucose had no significant effect on the particle size of LWE. However, the particle size of LWE was significantly increased when the concentration reached 0.04 g/mL, and the largest size was at 0.08 g/mL, which could be 2.6 times larger than the control. From the analysis of particle size distribution, it could be seen that the proportion of 10 μm particle distribution was increased by the addition of glucose, which might be attributed to the thermodynamic incompatibility effect; consequently, the hydrophobic interaction between protein particles was strengthened and ultimately caused the aggregation [45].

#### 3.2.2. Turbidity

**Figure 5:**
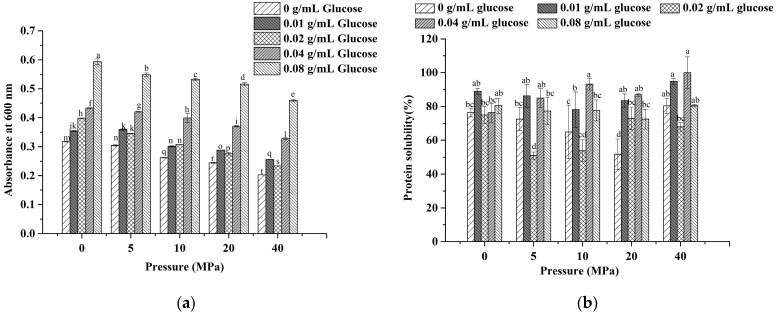
The turbidity (**a**) and solubility (**b**) of LWE under different homogenization pressure treatment and glucose concentrations. Note: The turbidity was defined by absorbance at 600 nm. Significant differences were indicated with different letters (*p* < 0.05).

It was demonstrated that the turbidity of LWE gradually decreased with the increase in the homogenization pressure and reached its lowest at 40 MPa when the glucose concentration was constant (Figure 5a). These results showed that homogenization reduced the turbidity of LWE. It might be the reason that the decreasing of protein particle size and the enhancement in the electrostatic repulsion between proteins triggered by higher pressure, make their droplets difficult to aggregate (Figure 4). The turbidity of LWE was increased with the increased glucose concentration when the homogenization pressure was constant. The glucose-induced emptying interactions between proteins resulted in the droplets around glucose attracting each other to aggregate, and the strength of interaction was linearly correlated with the concentration [46]. Overall, the turbidity of LWE was the highest at 0.08 g/mL glucose under 0 MPa and the lowest at 0 g/mL glucose under 40 MPa.

#### 3.2.3. Solubility

Protein plays a remarkable role in food in the form of protein-solvent. Therefore its solubility is one of the important characteristics of processing and production [47]. Many studies have demonstrated that the structure of the protein, the formation of the hydration layer, and the strength of the electrostatic interaction are all critical factors impacting protein solubility [48,49]. The results showed that the solubility was significantly decreased (*p* < 0.05) under the two treatments with 0.02 g/mL of glucose at 5 MPa and 0 g/mL of glucose at 20 MPa, while it was increased (*p* < 0.05) under three treatment conditions with 0.04 g/mL of glucose at 10 MPa, 0.01 g/mL of glucose at 40 MPa, and 0.04 g/mL of glucose at 40 MPa (Figure 5b).

Moreover, it was shown that there was no significant change in solubility with the increase in glucose concentration, and the general trend was upward. More oxygen atoms and hydrogen atoms from glucose are grafted on the proteins, and the hydrogen bonding and hydration between proteins and water molecules are enhanced accordingly [50]. When the pressure was at a lower level, the protein structure in LWE became partially unfolded by homogenization, and their internal hydrophobic groups were more exposed and distributed on the surface (Figure 1 and Figure 2). Accordingly, the solubility of the samples treated below 10 MPa observably reduced. When the pressure was over 10 MPa, the solubility was markedly increased. Actually, previous studies suggested that the high-speed shearing of homogenization prevented protein aggregation by blocking the formation of hydrogen bonds and affecting their surface charge states [8,51]. Moreover, the particle size of LWE was turned to be smaller by homogenization, which could improve the solubility of protein by strengthening the contact with water.

### 3.3. Processing Properties of LWE

#### 3.3.1. Foaming Properties

**Figure 6:**
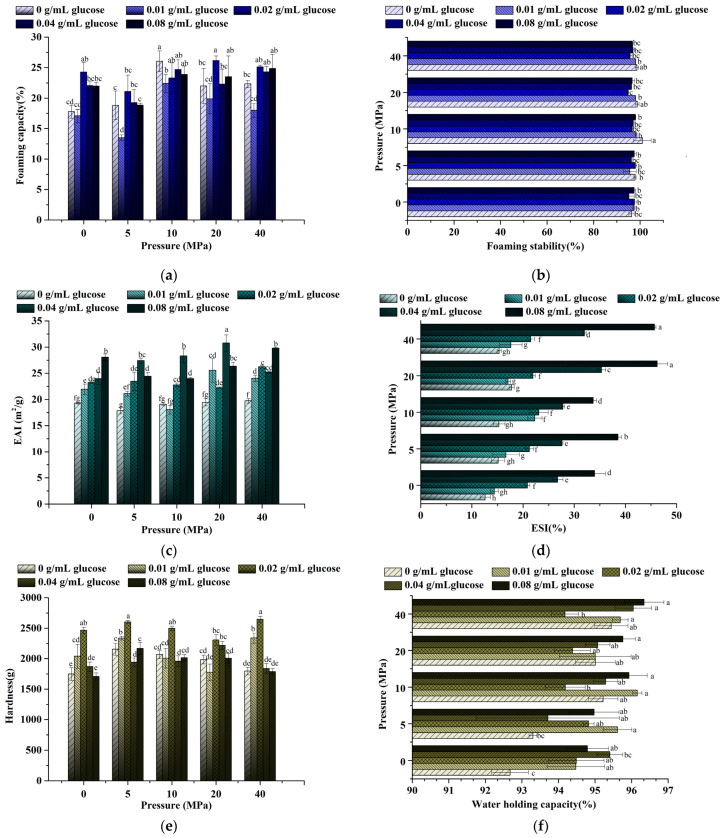
The processing properties of LWE under different homogenization pressure and glucose concentrations. Figures in turn are foaming capacity (**a**), foaming stability (**b**), emulsifying capacity (**c**), emulsifying stability (**d**), gel hardness, (**e**) and WHC (**f**). Significant differences were indicated with different letters (*p* < 0.05).

Many studies have shown that foam generation is influenced by the protein solubility, the surface tension of the gas-liquid interface [51], the liquid viscosity, and the absorbability of the protein at the phase interface [52]. It was shown that the foamability was the strongest under the condition of 0.02 g/mL glucose at 20 MPa (*p* < 0.05), which was 47.57 ± 5.1% higher than the control (Figure 6a). Except for the conditions of 0.01 g/mL glucose at 0 MPa and 0.01 g/mL glucose at 5 MPa, the foamability of LWE in other conditions was all enhanced.

Regarding homogenization, the foamability of the treated group was reduced at 5 MPa except for the LWE without glucose. When the pressure was increased to 5 MPa, the decrease in foamability corresponded to the decrease in solubility (Figure 5b). Meanwhile, the lipids competed with proteins for adsorption at the gas-liquid interface, and thereby the protein concentration in the interface was reduced [53]. The foamability was increased when the pressure reached 10 MPa. It was found that the protein structure was unfolded appropriately, and nonpolar regions were exposed on the surface (Figure 1 and Figure 2). However, the particles in LWE were smaller (Figure 4) when pressure was higher (20–40 MPa), leading to migration to the gas-liquid interface easier and faster, followed by a reduction in interfacial tension, and the foamability obtained a certain rebound accordingly. On the other hand, the foamability also showed a V-shaped change trend as glucose concentration increased, and it was the lowest at 0.01 g/mL. The viscosity of the liquid was higher than the control when glucose was at a lower level, making it harder for the air to pass through the interface [54]. On the contrary, the foamability showed a significant rebound (*p* < 0.05) when the glucose concentration was more than 0.02 g/mL. The phenomenon could be explained by an increase in protein concentration at the interface, which was caused by mutual repulsion between the proteins and glucose when glucose concentration was at a higher level [55]. Accordingly, the increased foamability was due to the reduction in interfacial tension.

Foam stability refers to the ability of proteins to stabilize foam under the action of mechanical force and gravity. It was shown that the stability under the condition of 0 g/mL glucose at 10 MPa was dramatically improved (*p* < 0.05) (Figure 6b). The toughness of proteins was increased by unfolding, resulting in a thickness increment of the protein film formed on the foam surface.

#### 3.3.2. Emulsifying Properties

To maintain the stability of the emulsion, the hydrophilic and hydrophobic groups in the protein molecular structure are usually rearranged, and a thick film is formed on the surface of oil droplets [56]. The formation of an emulsion is both affected by the ability of the emulsifier to migrate to the interface and its adsorption strength at the interface [57,58]. Except for the samples without glucose addition and with 0.01 g/mL glucose at 10 MPa treatment, the EAI was improved significantly by higher pressure (20–40 MPa) and glucose (*p* < 0.05) (Figure 6c,d). Of which, the EAI was the highest at 0.04 g/mL glucose at 20 MPa (Figure 6c). Regarding homogenization, a downward trend in the EAI existed when pressure was at 0–10 MPa. It was probable that the protein hydrophobic regions were likely to be exposed (Figure 1 and Figure 2) and the solubility decreased. (Figure 5b) R. Marco-Molés reported that EAI of homogenized LWE was decreased with the increase of pressure (in the range from 0 to 250 MPa) [59]. However, the emulsibility of LWE was markedly enhanced in this work when the pressure was increased to 20 MPa–40 MPa. It may be probable that the range of homogenization pressure was different. Previous studies suggested that the relationship between protein unfolding degree and emulsibility is not linear, while moderate protein structure modification is more beneficial for emulsification. The moderate unfolding made it easier for the hydrophobic to be embedded into the oil layer, which was conducive to the formation of a protein film [60]. Furthermore, more low-density lipoproteins (LDLs) might be released from the yolk after homogenization. Benefiting from the characteristics of lipoproteins, the binding between protein and oil droplets could be enhanced by LDLs during emulsification [61].

The emulsibility of LWE was improved by glucose. With the increase in glucose concentration, EAI showed a significant increasing trend. The reasons could be explained as follows: more -OH was introduced by glucose, and the hydrophilicity of the protein was raised [28]. Simultaneously, the viscosity of the LWE was also increased by glucose. Given that, the protein could better adhere to the surface of droplets, and thereby stronger protein films were formed. The best emulsibility was shown at 0.04 g/mL glucose at 20 MPa, which verified the moderate protein modification state mentioned earlier. The protein was unfolded more properly at 0.04 g/mL glucose under 20 MPa when compared with the conditions (no glucose or 0.08 g/mL glucose, no homogenization, or 40 MPa). Its hydrophilicity and hydrophobicity were almost kept in a state of equilibrium [60], and the best emulsifying ability was shown in this condition, undoubtedly.

Both the homogenization and the glucose addition significantly enhanced the emulsification stability of the LWE (*p* < 0.05), and it reached its highest value under the condition of 0.08 g/mL glucose at 20 MPa, which was nearly four times higher than the control. The emulsification stability showed no remarkable change when the homogenization pressure was at a lower level (0–10 MPa) (Figure 6d), but it was enhanced as the pressure rose to a higher level (10–20 MPa). The enhancement might be that the protein structure was unfolded and better absorbed at the oil-water interface to maintain the emulsion stability. However, as the pressure further increased to 40 MPa, the stability was slightly weakened. It was well known that high pressure will lead to excessive exposure to the hydrophobic structure in protein, which causes the droplet’s agglutination to destroy the stability. Glucose had a more conspicuous effect on improving the emulsification stability in comparison with homogenization. The stability was enhanced with the increase in glucose concentration, especially at a high level (0.04–0.08 g/mL). It was reported that emulsions’ stability made from a mixture of protein and polysaccharides were not easily destroyed by flocculation [62]. Additionally, studies have found that saccharides will change the protein film thickness at the oil-water interface [63]. Therefore, the adhesion rate of the glucose to the protein increased, and the surface adsorption layer of oil droplets formed by the glucose–protein combination was thicker. In this way, the steric hindrance of the emulsifier increased [64], which led to the enhancement of repulsive interaction between the oil droplets. This phenomenon was also reflected in the related research on soy protein performed by Tran and Rousseau [65].

#### 3.3.3. Gelling Properties

The LWE could be heated to form a thermally induced gel [66]. The formation of the gel network was mainly affected by hydrophobic interaction, electrostatic interaction, and the formation of some covalent bonds between proteins [67]. Hardness is often defined as one of the important indicators to evaluate the quality of the gel. It was shown that a lower concentration of glucose (0–0.02 g/mL) and homogenization markedly improved the hardness of LWE gel (*p* < 0.05) (Figure 6e).

Regarding homogenization, the hardness of the LWE gel increased first and then decreased with the increase in the pressure when the pressure was less than 20 MPa. Once the pressure reached 40 MPa, the hardness with a low concentration of glucose (0.01–0.02 g/mL) increased remarkably (*p* < 0.05). The protein structure was properly unfolded when pressure was below 20 MPa. The hydrophobic structure was partially exposed and hydrophobic interaction was enhanced, which was conducive to the formation of the gel network. Moreover, LDLs were released from the egg yolk and distributed more evenly in LWE after homogenization, and it was proved to dominate the gelation of egg yolk [68]. The fat molecules could fill more voids in the gel structure of egg yolk and lead to a reinforcement of the protein gel network since LDLs could promote the interaction between oil droplets and proteins [69]. Studies have shown that the formation of more stable and permanent chemical cross-linking in the gel grid could be promoted by disulfide bonds [70]. Therefore, when the pressure is increased to 10–20 MPa, the decrease in hardness might be due to the breakage of disulfide bonds by high pressure (Figure 3). Interestingly, the hardness of some samples rebounded when the pressure increased to 40 MPa, which was caused by the change in particle size. The lipid and protein were evenly mixed, and the particles became smaller after shearing, which further reduced the steric hindrance of cross-linking and made it easier to form a uniform and compact network structure.

Regarding the addition of glucose, the changing trend of hardness was increased firstly and then decreased, and it reached a maximum at 0.02 g/mL. The results of free sulfhydryl content showed that more disulfide bonds were formed at 0.02 g/mL (Figure 3), resulting in the gel network more rigid. It was found that there was competitive hydration between glucose and protein, protein dehydration, as well as large-scale aggregation when the glucose concentration was higher (0.04–0.08 g/mL) [71]. Therefore, the gel was less uniform and more fragile (*p* < 0.05).

The WHC would be enhanced by both homogenization and glucose and reached the maximum at 0.08 g/mL glucose-40 MPa (Figure 6f), which might be related to the increase in the number of charged groups in protein. Lipids released from yolk also enhanced the homogeneity of the protein gel matrix, and there were some “pockets structure” that could bind large amounts of water, resulting in an increase in WHC [67].

## 4. Conclusions

Homogenized LWE had better performance on the processing properties after the glucose addition. Remarkable increments of 47.57 ± 5.1% and 66.79 ± 9.55% in foaming capacity (0.02 g/mL glucose at 20 MPa) and emulsifying capacity (0.04 g/mL glucose at 20 MPa) were found in the treated LWE, respectively, and the generated LWE gel under the condition of 0.02 g/mL glucose at 40 MPa had high hardness and strong WHC. In fact, under these treatment conditions, protein structure and physicochemical properties were modified to a more appropriate degree, including moderate protein unfolding, surface hydrophobicity, and particle size.

Glucose was added into LWE before homogenization for flavor improvement. However, processing properties might be worse under some improper treatment conditions. In this work, it was found that both protein structure and physicochemical properties were affected by glucose and homogenization, and the proper glucose addition improved the quality of LWE under homogenization treatment. Furthermore, the optimal glucose concentration and treatment pressure (0.02–0.04 g/mL and 20–40 MPa) could be referenced to improve the processing properties and overall quality of industrial LWE products.

## Data Availability

The data presented in this study are available on request from the corresponding author.

## Abbreviations

8-Anilino-1-naphthalenesulfonic acid (ANS); absorbance units (AU); bovine serum albumin (BSA); 5,5′-Dithiobis-(2-nitrobenzoic acid) (DTNB); emulsifying stability index (EAI); ethylene diamine tetraacetic acid (EDTA); emulsifying activity index (ESI); foaming capacity (FC); foaming stability (FS); low-density lipoproteins (LDLs); liquid whole eggs (LWE); phosphate buffered saline (PBS); particle size distribution (PSD); sodium dodecyl sulfate (SDS); Tris-(hydroxymethyl)-aminomethane (Tris); water holding capacity (WHC).
